# Evaluating analytical quality in clinical biochemistry laboratory using Six Sigma

**DOI:** 10.11613/BM.2018.020904

**Published:** 2018-06-15

**Authors:** Xuehui Mao, Jing Shao, Bingchang Zhang, Yong Wang

**Affiliations:** Shandong Provincial Hospital Affiliated to Shandong University, Jinan, Shandong, People’s Republic of China

**Keywords:** Sigma metrics, total allowable error, quality control, bias

## Abstract

**Introduction:**

In recent years, Six Sigma metrics has became the hotspot in all trades and professions, which contributes a general procedure to explain the performance on sigma scale. Nowadays, many large companies, such as General Healthcare, Siemens, *etc*., have applied Six Sigma to clinical medicine and achieved satisfactory results. In this paper, we aim to evaluate the process performance of our laboratory by using Sigma metrics, thereby choosing the correct analytical quality control approach for each parameter.

**Materials and methods:**

This study was conducted in the clinical chemistry laboratory of Shandong Provincial Hospital. The five-months data of internal quality control were harvested for the parameters: amylase (AMY), lactate dehydrogenase (LD), potassium, total bilirubin (TBIL), triglyceride, aspartate aminotransferase (AST), uric acid, high density lipoprotein-cholesterol (HDL-C), alanine aminotransferase (ALT), urea, sodium, chlorine, magnesium, alkaline phosphatase (ALP), creatinine (CRE), total protein, creatine kinase (CK), total cholesterol, glucose (GLU), albumin (ALB). Sigma metrics were calculated using total allowable error, precision and percent bias for the above-mentioned parameters.

**Results:**

Sigma values of urea and sodium were below 3. Sigma values of total protein, CK, total cholesterol, GLU and ALB were in the range of 3 to 6. Sigma values of AMY, uric acid, HDL-C, TBIL, ALT, triglyceride, AST, ALP and CRE were more than 6.

**Conclusion:**

Amylase was the best performer with a Sigma metrics value of 19.93, while sodium had the least average sigma values of 2.23. Actions should be taken to improve method performance for these parameters with sigma below 3.

## Introduction

In the health care sector, reports from clinical laboratory are of vital importance in decision-making. Around 70% of the patient-related decision is based on the clinical laboratory (*1*). According to the statistics, the estimated error rates in the three phases of total testing procedure including pre-analytical, analytical and post-analytical phase are 30 - 75%, 4 - 30% and 9 - 55%, respectively (*2*). Hence, stringent quality control in clinical laboratory is essential for patient care.

Quality control (QC) is the foundation for assuring accuracy and precision of the analytical process and detection of immediate error. It involves two basic types of schemes - external QC (EQC) and internal QC (IQC) (*3*). External QC analyzes and reports of control samples given by an external agency once a month, while IQC ensures continuous monitoring of the analytical system by conducted everyday. Therefore, it guarantees the results are reliable before they are released. In 1981, Dr. James O. Westgard proposed several statistical process control rules used with Levey-Jennings chart for evaluating QC performance (*2*). However, both EQC and IQC cannot be used for assessing exact number of defects or errors in laboratory.

The problem mentioned above can be solved by employing sigma (σ) metrics in the laboratory, which provides a more quantitative work frame for assessing process performance and creates a scientific basis for designing an appropriate QC strategy. In the year 2000 and 2001 respectively, Nevalainen *et al.* and Westgard were the first to evaluate laboratory’s performance on six sigma scale (*4*, *5*). Sigma value can be calculated with known total allowable error, estimated accuracy (bias) and precision (CV%) (*6*). According to Nevalainen *et al.,* “average products, regardless of their complexity, have a quality performance value of about 4σ. The best, or ‘world class quality,’ products have a level of performance of 6σ” (*4*). Thus, we are able to assess the quality of our laboratory testing processes by Six Sigma metrics and choose suitable QC rules needed. Six Sigma metrics can serve as a self-assessment method in guiding clinical laboratory to make QC strategy and plan QC frequency. It’s very helpful to implement this metrics into clinical laboratory daily analytical processes in order to produce accurate test results. Thus the aim of our study was to calculate Sigma metrics of clinical chemistry analytes and make the appreciate quality control strategy.

## Materials and methods

### Study design

This study was conducted in the clinical chemistry laboratory of Shandong Provincial Hospital. The five-months data of internal quality control were harvested for the following parameters: amylase (AMY), lactate dehydrogenase (LD), potassium, total bilirubin (TBIL), triglyceride, aspartate aminotransferase (AST), uric acid, high density lipoprotein-cholesterol (HDL-C), alanine aminotransferase (ALT), urea, sodium, chlorine, magnesium, alkaline phosphatase (ALP), creatinine (CRE), total protein, creatine kinase (CK), total cholesterol, glucose (GLU), albumin (ALB). Sigma metrics were calculated using total allowable error, CV and percent bias for the above-mentioned parameters. Two levels of clinical chemistry controls (581SD170, Lyphochek™ Assayed Chemistry Control, Bio-Rad, Marnes-la-Coquette, France) were used for each parameter and tested twice a day at 7:00 am and 11:00 am. External QC and IQC data of 20 analytes from April 2017 to August 2017 were analysed. All tests were run on Beckman-Coulter AU 5800 (Beckman Coulter, Brea, USA) according to the manufacturer’s recommendations.

### Statistical analysis

Total allowable error (TEa) indicates allowable difference from the true values. The TEa values of various parameters were taken from Clinical Laboratories Improvement Act (CLIA) guidelines (*7*).

Bias is the systematic difference between the results obtained by the lab’s test method and the results obtained from an accepted reference method. Bias was computed from external quality assurance records with following formula: Bias = (Lab mean - Group mean) x 100 / Group mean. The mean bias was used for sigma value calculation.

Coefficient of variance (CV) is the analytical coefficient of variation of the test method. It was determined from the calculated laboratory mean and calculated standard deviation procured from 5 months of IQC data: CV% = (standard deviation / laboratory mean) x 100%.

The Sigma metrics was calculated with following formula: Sigma metrics = (TEa - Bias%) / CV%.

## Results

We calculated Bias, CV% and Sigma metrics for the 20 parameters (Table 1). Each control had two levels and was tested twice a day at 7:00 am and 11:00 am. The results are listed in Table 1 and 2. For parameters blood urea and sodium, sigma values were below 3. For parameters: total protein, CK, cholesterol total, GLU and ALB, sigma values were in the range of 3 to 6. For parameters: AMY, uric acid, HDL-C, TBIL, ALT, triglyceride, AST, ALP and CRE, the sigma metrics were higher than 6. Among these analytes, AMY was the best performer with an average sigma metrics value of 19.93, while sodium had the least average sigma values of 2.23.

**Table 1:** Total allowable error (TEa), precision (CV) bias, and Sigma value for the controls tested

**Parameter**	**TEa**	**Average Bias (%)**	**Level 1**			**Level 2**	
**CV**		**Sigma**	**CV**	**Sigma**
ALB	10	3.82	1.62		3.81	1.68	3.68
ALP	30	2.84	3.98		6.82	3.8	7.15
ALT	20	4.66	1.97		7.79	1.65	9.30
AMY	30	3.29	1.34		19.93	1.36	19.64
AST	20	0.12	2.81		7.07	2.03	9.79
CK	30	1.58	5.31		5.35	5.12	5.55
CRE	15	0.11	2.43		6.13	2.15	6.93
Cholesterol total	10	1.38	1.66		5.19	1.59	5.42
GLU	10	2.38	1.94		3.93	1.86	4.10
HDL-C	30	8.71	2.31		9.22	2.26	9.42
LD	20	2.47	2.94		5.96	2.39	7.33
Potassium	6	1.81	1.30		3.2	1.62	2.59
Total protein	10	3.12	1.32		5.21	1.15	5.98
TBIL	20	1.1	2.09		9.04	1.37	13.80
Triglyceride	25	4.26		2.68	7.74	1.88	11.03
Uric acid	17	4.54		1.30	9.58	1.31	9.51
Blood urea	9	1.59		2.32	3.19	2.61	2.8
Sodium	5	1.80		1.21	2.64	1.43	2.23
Chlorine	5	1.13		1.12	3.46	1.34	2.88
Magnesium	25	7.62		2.58	6.74	3.01	5.77
ALB – albumin. AMY – amylase. AST - aspartate aminotransferase. ALT - alanine aminotransferase. ALP - alkaline phosphatase. HDL-C - high density lipoprotein-cholesterol. CRE – creatinine. CK - creatine kinase. GLU – glucose. LD - lactate dehydrogenase. TBIL - total bilirubin.							

**Table 2:** Quality control strategy for future works

**Sigma metrics**	**Parameters**	**Levels of control**	**Measurements**	**Westgard rules**
> 6	AMY, AST, ALP, ALT, CRE, HDL-C, TBIL, Triglyceride, Uric acid	2	1	1_3S_
4 - 6	CK, Cholesterol total, GLU, LD, Total protein	2	1	1_2.5S_
3 - 4	ALB	2	2	1_3S_/2_2S_/R_4S_/4_1S_
< 3	Blood urea, Chlorine, Sodium, Potassium	3	2	1_3S_/2_2S_/R_4S_/4_1S_
ALB – albumin. AMY – amylase. AST - aspartate aminotransferase. ALT - alanine aminotransferase. ALP - alkaline phosphatase . HDL-C - high density lipoprotein-cholesterol. CRE – creatinine. CK - creatine kinase. GLU – glucose. LD - lactate dehydrogenase. TBIL - total bilirubin.				

## Discussion

In this study, we analysed 20 parameters over a period of 5 months. Six sigma improves the quality of process outputs by analysing and abolishing the source of defects and reducing variability in manufacturing and business practices. In terms of clinical laboratory, the identification of test with low sigma values (< 3σ) indicate that actions should be taken to improve analytic quality or the lab should use alternate methods and reagents (*8*, *9*).

According to our results, AMY was the best performer with an average sigma metrics value of 19.93, while sodium had the least average sigma values of 2.23. For parameters blood urea and sodium, sigma values were below 3. Method performance must be improved before the method can be used for further production and three levels of QC with a 1_3S_/2_2S_/R_4S_/4_1S_ rule should be taken twice a day. We should strictly follow internal QC and Westgard multi rules and pay special attention to these parameters. Besides, sigma values may increase by upgrading analysers and better methodologies. For parameters like ALB with 3-4σ, a 1_3S_/2_2S_/R_4S_/4_1S_ rule should be used. For parameters like total protein, CK, total cholesterol and GLU, sigma values were in the range of 4 to 6, QC monitoring have to be done with a 1_2.5S_ rule. For parameters: AMY, uric acid, HDL-C, TBIL, ALT, triglyceride, AST, ALP and CRE, the sigma metrics were higher than 6. This existing QC protocol with a 1_3S_ rule need no change and the testing results can be released directly (*10*). Variations between our statistical data and others were due to the difference in QC samples as well as instrument and method differences. It is very clear that a laboratory with high sigma tests has an easy time for designing procedure for implementation. These data showed us that ideal methodologies are being used in our biochemistry laboratory. For these parameters with low sigma values, we will use the optimized QC strategy for future works.

Sigma metrics was calcuated by using TEa, bias and CV. This method is not the ideal way, but the practical way. The ideal ways include using reference materials or comparison with reference methods. The results from clinical laboratory have a large impact on patients’ lives. However, there is no particular guideline of rules implementation based on the performance of each test and method, which can cause increase of false rejection and waste of control samples for testing laboratory. Thus, choosing a specific QC procedure will minimize the false rejection and maximize the error detection (*3*). Clinical laboratory focus on producing accurate test results, so it make sense to implement six sigma metrics into their daily analytical processes. Six Sigma metrics can serve as a self assessment method in guiding clinical laboratory to make QC strategy and plan QC frequency. It’s very helpful to implement this metrics into our laboratory daily analytical processes in order to produce accurate test results.
